# Actinomycetoma with systemic features: A warning sign for immunosuppression?

**DOI:** 10.1371/journal.pntd.0008865

**Published:** 2020-12-03

**Authors:** Rita Fernanda Cortez de Almeida, Roberta Espírito Santo Correia, Andréa Gina Varón, Janice Mery Chicarino de Oliveira Coelho, Ana Paola de Oliveira, Maria Cristina Silva Lourenço, Erica Aparecida dos Santos Ribeiro da Silva, Emilyn Costa Conceição, Cristiane da Cruz Lamas, Dayvison Francis Saraiva Freitas

**Affiliations:** 1 Department of Dermatology, Bonsucesso Federal Hospital, Rio de Janeiro, Brazil; 2 Evandro Chagas National Institute of Infectious Diseases, Oswaldo Cruz Foundation, Rio de Janeiro, Brazil; 3 Grande Rio University, Rio de Janeiro, Brazil; UNITED STATES

**Key Learning Points**

- Actinomycetoma is a chronic infection usually affecting the subcutaneous tissues of the lower limbs.
- If systemic signs accompany the localized infection, an underlying immunosuppressive condition should be considered.
- The inclusion of mycetoma in the WHO list of neglected diseases will hopefully increase awareness of this neglected disease.

## Case presentation

A 32-year-old man was referred to our Dermatology Outpatient Unit with the clinical suspicion of sporotrichosis. At the age of 8, he had suffered a penetrating injury to his right foot, which remained as a painless nodule for years. One year before his outpatient appointment, the nodule progressively increased in size and ulcerated, and in the last 6 months, he began to experience night sweats, a low-grade fever, a 12-kg weight loss, and malaise.

**Fig 1:**
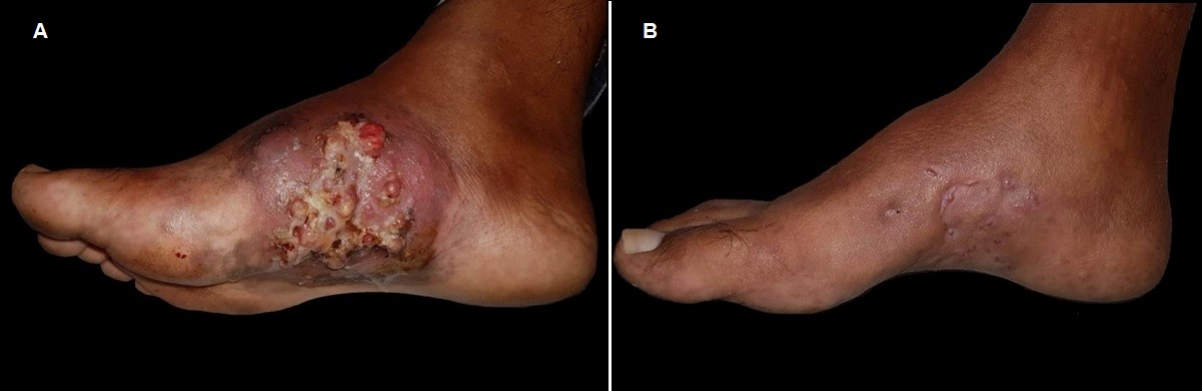
Clinical image from a 32-year-old patient with a long-term history of indolent actinomycetoma with rapid progression and systemic signs. **(A)** Image obtained on the first day shows tumefaction with multiple nodules, fistulae, and purulent discharge on the medial arch of the right foot. **(B)** Image obtained following 7 months on cotrimoxazole therapy shows the actinomycetoma healing.

**Fig 2:**
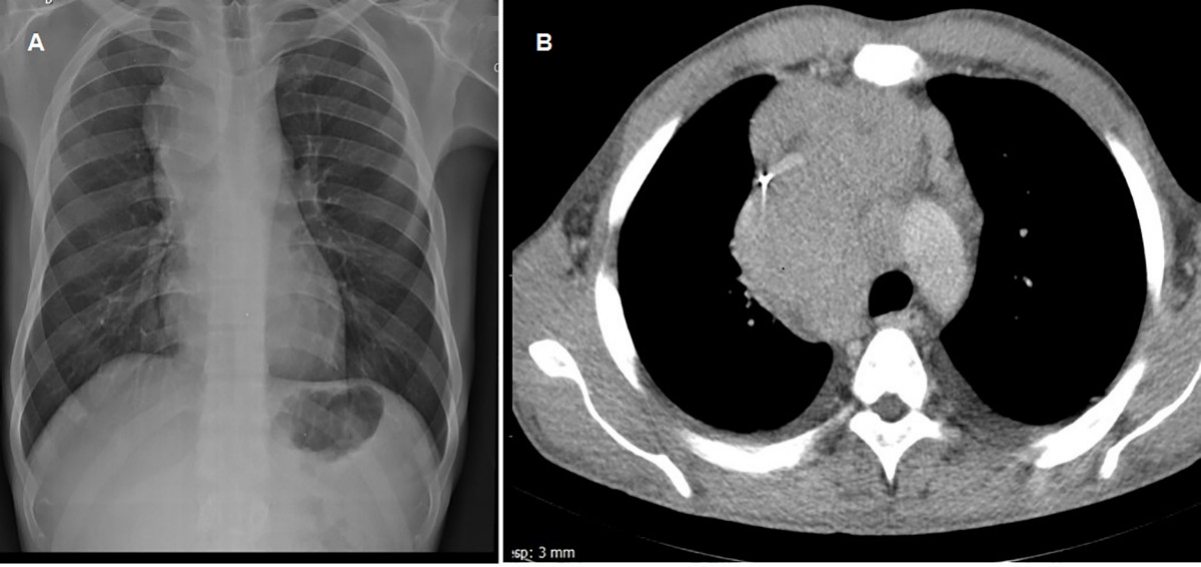
Chest imaging from a 32-year-old patient with a long-term history of indolent actinomycetoma with rapid progression and systemic signs. **(A)** Chest X-rays demonstrate mediastinal enlargement. **(B)** Computed tomography scan of the chest obtained on the admission shows enlarged mediastinal lymph node, one of them with a central hypodensity and the presence of minimal bilateral pleural effusion.

**Fig 3:**
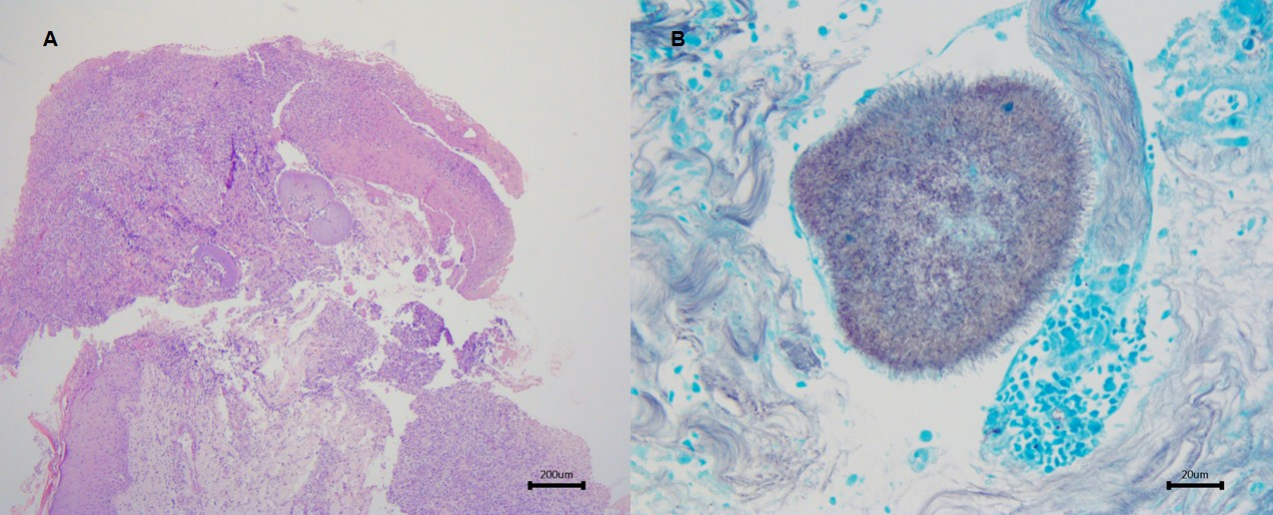
Histopathological features of the skin biopsy from a 32-year-old patient with a long-term history of indolent actinomycetoma with rapid progression and systemic signs. **(A)** Ulcerated skin shows filamentous grain intermingled in inflammatory infiltrate. H&E. **(B)** Numerous radiated filaments emerge from the grain. Grocott methenamine silver stain. H&E, hematoxylin and eosin.

**Fig 4:**
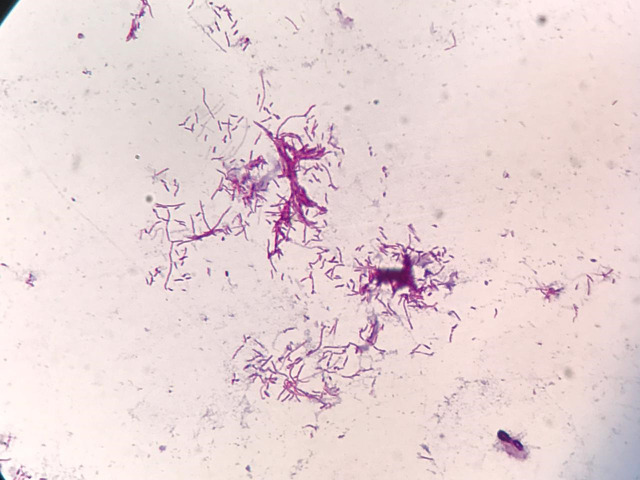
Culture of skin tissue obtained by punch biopsy from a 32-year-old patient with a long-term history of indolent actinomycetoma with rapid progression and systemic signs. The image shows gram-positive, partially acid-fast, fine, branching filamentous bacilli.

On examination, he had a foul-smelling and painful tumefaction with multiple nodules, fistulae, and purulent discharge, on the medial arch of his right foot (Fig 1A). He was pyrexial (37.9°C), tachycardic (heart rate 126), but otherwise, other physical signs were unremarkable. The differential diagnoses considered for the skin lesion were dermatofibrosarcoma protuberans, amelanotic melanoma, Kaposi’s sarcoma, and actinomycetoma. Exams were performed (biochemistry screen, a full blood count, and a chest X-ray). Amoxicillin clavulanate was initiated, and a punch biopsy of the lesion was done. The patient remained febrile and did not gain weight despite 3 weeks of antibiotics. Chest X-ray showed an enlarged mediastinum (Fig 2A). He was admitted to hospital for further investigation and treatment. Full blood count showed hemoglobin of 5.7 g/dL, 12,250 white blood cells/mm^3^, and lymphocytes of 2,082 cells/mm^3^; C-reactive protein was 21.78 mg/L (reference value < 0.3); liver and kidney function tests were normal. Blood cultures and serological tests for HIV, toxoplasmosis, syphilis, cytomegalovirus, and hepatitis B and C were all negative. Because he was unwell and hypotensive on admission, empirical treatment with cotrimoxazole and imipenem was started. Histopathology of the skin biopsy revealed filamentous grain-producing bacteria in hematoxylin and eosin (H&E) stain (Fig 3A). The bacteria stained positive by the Grocott methenamine silver (Fig 3B), Wade, and Gram stains, suggestive of *Nocardia* spp. Cultures of this fragment in Thayer-Martin and agar-chocolate media showed growth of gram-positive, partially acid-fast bacilli identified as *Nocardia* spp. (Fig 4) by phenotypic screening tests, such as growth in lysozyme and detection of filamentous growth on coverslip. Molecular identification of the species was obtained by sequencing the polymerase chain reaction product for the hsp65 gene, according to Telenti and colleagues with specific primers (TB11,5′-ACCAACGATGGTGTGTCCAT-3') and (TB12,5′-CTTGTCGAACCGCATACCCT-3′) resulting in the diagnosis of the species *Nocardia nova* [1]. There were no radiographic signs of bone involvement of the right foot. As part of the investigation of the patient’s general symptoms, which are unusual in actinomycetoma, along with the evidence of mediastinal enlargement seen in the chest X-ray, a computed tomography (CT) scan of the chest was ordered and showed enlarged mediastinal lymph nodes, one with a central hypodensity, and the presence of minimal bilateral pleural effusion (Fig 2B). CT scan of the abdomen showed hepatomegaly and a slightly enlarged spleen. As the patient presented B symptoms and mediastinal lymphadenopathy, a diagnosis of localized actinomycetoma with rapid progression due to an underlying cancer was our main suspicion, followed by a concern of a possible disseminated nocardiosis, which was never documented.

A bone marrow biopsy was done, and microscopy showed 90% of hematopoietic cellularity, normoblastic erythroid series, hyperplastic granulocyte series with normal maturation, and megakaryocytes with normal morphology. There were no granulomas, neoplasia, fungi, or mycobacteria. Bone marrow cultures were negative. A CT-guided mediastinal lymph node biopsy was done, and microscopy showed positive immunohistochemistry for CD30, CD15, and PAX15 and negative for CD3, CD20, and LCA, a classic Nodular Sclerosing Hodgkin’s lymphoma pattern.

**Fig 5:**
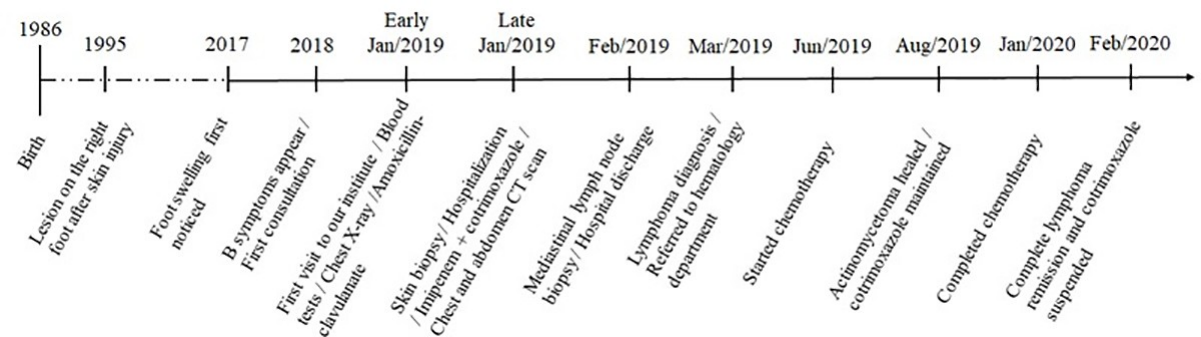
Timeline with some relevant clinical facts.

Imipenem was stopped after 19 days of treatment; cotrimoxazole was continued and the skin lesion improved (Fig 1B). The patient was referred to a hematology department for the lymphoma treatment, with adriamycin, vinblastine, and dacarbazine. At the 7-month follow-up visit with us, the actinomycetoma was healed; his general health condition consistently improved, he gained 13 kg, and had no fever nor night sweats. The chemotherapy sessions were complete on January 22, 2020, when complete remission was considered, after a positron emission tomography (PET)-CT scan. Cotrimoxazole treatment was maintained until February 13, 2020. A timeline figure helps illustrate some clinical facts (Fig 5*)*.

## Case discussion

Mycetoma is a neglected tropical disease; when caused by bacteria, it is named actinomycetoma, and when by fungi, eumycetoma. *Nocardia* spp. are aerobic filamentous bacteria first recognized as pathogens by Nocard in 1888; they are the main causative agents of actinomycetoma [2–4]. This genus comprises catalase-positive gram-positive rods, which are acid-fast. It produces pale, white, or yellow grains and can be grown on Sabouraud agar [5]. *Nocardia* spp. have a worldwide distribution, being found in fresh and saltwater, decaying vegetation, and soil. *N*. *nova*, nowadays considered a complex of species, is associated with rare cases of actinomycetoma [6]. Infections occur by inhalation or skin trauma, usually resulting in localized infections, mostly in feet, which affect both immunocompetent and immunocompromised patients [3,6]. The typical findings are a triad, which involves painless (or oligosymptomatic) subcutaneous masses, multiple fistulae and grains of different colors, sizes, and consistency, that drain from these fistulae [2,5,6]. This triad points to a few differential diagnoses: botryomycosis, eumycetoma, or actinomycetoma, according to the causative agent (*Staphylococcus* spp. or other bacteria, filamentous fungi, or aerobic filamentous bacteria, respectively) [7]. The diagnosis of *Nocardia* spp. infection may go underrecognized because of its relative rarity and its slow growth. Immunosuppressive conditions related to nocardiosis are solid organ and bone marrow transplantation, chronic renal failure, chronic lung disease, HIV/AIDS, cancer, and hematological malignancies. Hematological patients are an at-risk group for nocardiosis due to the intrinsic and therapy-related immunodeficiency [7–8].

We report a case of actinomycetoma with systemic signs, which lead us to investigate an underlying immunosuppression, since such signs are uncommon in this deep and usually localized infection. A literature search of PubMed with the keywords “nocardiosis” and “lymphoma” resulted in 115 articles, of which 10 were cases of primary cutaneous nocardiosis related to hematological malignancies (Table 1) [9–18]. Most cases were reported from the United States; all presented *Nocardia* spp. infection involving the skin and subcutaneous tissues as a complication of the underlying hematologic condition (after 2 to 24 months, when specified). There is the possibility of latent infection, as in our case, or infection that developed after the malignancy. In the present case, we believe that *N*. *nova* had been under immune control, for years, but because of the recent lymphoma-related immunosuppression, control was lost, and the cutaneous lesion flourished. A possible mechanism for the reactivation of the infection is that the anti-inflammatory cytokines interleukin 10 (IL-10) and transforming growth factor beta (TGF-β), produced by Hodgkin’s lymphoma cells, have inhibited the activity of phagocytic cells, thus allowing the proliferation of the *N*. *nova* [19].

**Table 1:** Summary of clinical cases (n: 10) of primary cutaneous nocardiosis and hematologic malignancies reported to date.

Reference	Year	Sex (age, y)	Site of infection	Months between malignancy and onset of nocardiosis	Hematologic malignancy	Country
[9]	1976	M (40)	Chest and axilla	7	HL	US
[10]	1981	M (59)	Arm	16–19	Histiocytic lymphoma	US
[11]	1984	F (65)	Forearm	6	Lymphosarcoma	Israel
[12]	1985	M (19)	Thigh	7	Small cleaved-cell NHL	Canada
[13]	1985	M (47)	Knee	Unspecified	T-cell leukemia	Japan
[14]	1988	M (74)	Hand	2	NHL	US
[15]	1989	F (73)	Axilla, arm, and thigh	24	Diffuse lymphoblastic NHL	England
[16]	1992	M (49)	Arm	24	Intestinal lymphoma	Israel
[17]	2006	F (65)	Mid-thigh	Unspecified	Infradiaphragmatic HL	US
[18]	2016	M (76)	Forearm	24	Marginal zone lymphoma	France

F, female; HL, Hodgkin’s lymphoma; M, male; NHL, non-Hodgkin’s lymphoma; US, United States of America.

The molecular identification of *N*. *nova* was late, but it did not compromise the case handling. It is important to emphasize the delay in the diagnosis (around 1 year), which we attribute not only to the limited access of the poorer population to the health system, but also to a low level of suspicion for the diagnosis of actinomycetoma and to an underlying condition. Hopefully, with the inclusion of mycetoma in the WHO list of neglected tropical diseases, this situation in Brazil and in other developing countries may change for the better.

Our strengths are to have been able to clinically suspect and confirm the patient’s diagnosis, as we are a reference institution for infectious diseases, with a good standard routine Microbiology and Pathology laboratories. Secondly, prompt referral to a Cancer Institute led to the diagnosis and treatment of the lymphoma. This case describes the rapid progression of an over 2-decades-old localized actinomycetoma lesion due to an underlying immunosuppressive condition.

## Ethics statement

All procedures performed were in accordance with the ethical standards laid down in the 1964 Declaration of Helsinki and its later amendments, as well as the Brazilian ethical standards—Resolution (CNS 466/12). Written consent has been obtained from the patient after a full explanation of the purpose and nature of all procedures used. The Institutional Review Board approved the case report under the number 27466619.8.0000.5262.
